# Supplementary material for: WordCommentsAnalyzer: A windows software tool for qualitative research
Source: F1000Res. 2018 Sep 4;7:536. Originally published 2018 May 3. [Version 2] doi: 10.12688/f1000research.14819.2 (PMC6137411; doi:10.12688/f1000research.14819.2)
Supplement: Supplementary file 3 [file f1000research-7-17614-s0002.tgz › b2a79496-4ada-47c4-9682-a99ce867836d.zip!/Eight Iranian Health Journals/Yafteh _51.docx]

© Yafteh  . This work is licensed under CC BY 4.0 (https://creativecommons.org/licenses/by/4.0/).

Volume & Issue: دوره 8 - شماره 2

Title: تعیین انواع اسکولیوز براساس محل آناتومیکی قله انحناء در سنین بلوغ 15-11 ساله مدارس راهنمایی استان اهواز

http://yafte.lums.ac.ir/article-1-1006-fa.html

مقدمه: اسکولیوز به معنی انحنای جانبی ستون فقرات است. این عارضه بسته به سن تشخیص به سه دسته کودکی، نوجوانی و جوانی تقسیم می شود. پیشرفت انحناء به سن، جنس و بلوغ اسکلتی بیمار بستگی دارد. برای توقف و تصحیح اسکولیوز در افرادی که رشد اسکلتی آنان کامل نشده است و انحنای طرفی ستون فقرات تا 20 درجه کوب دارند، کنترل و پیگیری انجام می شود، کسانی که انحنای 40-20 درجه کوب دارند، با پوشیدن بریس مداوا می شوند و افرادی که دارای انحنای 50-40 درجه کوب هستند، عمل جراحی توصیه می شود. این بررسی به منظور شناخت به موقع اسکولیوز، تعیین میزان شیوع و انواع آن بر اساس قله آناتومیکی انحناء در ستون فقرات انجام شده است تا با شناسایی نمونه های مبتلا و معرفی آنان به پزشک متخصص جهت پی گیری و درمان، از عوارض بعدی جلوگیری شود. نتیجه گیری: اسکولیوز علاوه بر ایجاد نقیصه ظاهری جسمی، برکارایی ارگان های داخلی مهمی مانند قلب و ریه نیز اثر دارد و گاهی همراه با ناهنجاری های کلیوی است. شناسایی این افراد و پی گیری آنان از گسترش صدمات جبران ناپذیر بعدی جلوگیری می نماید. با غربالگری مدارس برای یافتن نمونه های مبتلا در سنین کودکی، نوجوانی و جوانی با تشخیص به موقع و جلوگیری از پیشرفت آن می توان در سلامت جامعه نقش مهمی داشت. یافته ها: در این مطالعه 42 نفر(7/1%) از جمعیت مورد مطالعه مبتلا به اسکولیوز بودند که 28 نفر مؤنث و 14 نفر مذکر بودند که نسبت دختران به پسران 2 به 1 بود. در 7/85% افراد مونث مبتلا به اسکولیوز زاویه انحناء کمتر از 20 درجه کوب بود و در 3/14% آنها زاویه انحناء 30-20 درجه کوب محاسبه شد و زاویه انحناء بیشتر از 30 درجه کوب در 3/14% مشاهده شد ولی در نمونه های پسر مبتلا به اسکولیوز زاویه انحناء کمتر از 20 درجه کوب در 8/77%، با زاویه انحناء 30-20 درجه کوب در 1/11% و با زاویه انحناء بیشتر از 30 درجه کوب در 1/11% مشاهد شد. مواد و روش ها: در این بررسی که طی سالهای 83-1382 انجام شد، تعداد 2433 نمونه، شامل 1400 نفر دختر و 1033 نفر پسر به صورت تصادفی خوشه ای از مدارس راهنمایی شهرستان اهواز انتخاب شدند و ستون فقرات آنان با مشاهده مستقیم، علامت گذاری خار مهره ها با ماژیک و با استفاده از شاقول مورد معاینه قرار گرفت و وجود هرگونه انحناء به طرفین، اسکولیوز قلمداد شد و برای بررسی بیشتر به پزشک متخصص ارجاع داده شد.

Title: بررسی اثرات درمانی سرکه و آبغوره روی هایپرلیپیدمی در مقایسه با لواستاتین

http://yafte.lums.ac.ir/article-1-1007-fa.html

مقدمه: هایپرلیپیدمی و خصوصاً افزایش کلسترول خون یک ریسک فاکتور مهم بیماریهای قلب و عروق می باشد. انواع مختلفی از داروها، با عوارض فراوان جهت درمان هایپرلیپیدمی وجود دارد.یک دسته از رایج ترین آنها در درمان هایپرکلسترولمی استاتین ها و خصوصا لواستاتین می باشد. سرکه سیب و آبغوره نیز به عنوان دو ترکیب طبیعی در رژیم غذایی می توانند در درمان هایپرلیپیدمی مؤثر باشند.هدف از انجام این مطالعه تعیین کارایی و اثرات درمانی سرکه سیب و آبغوره در مقایسه با لواستاتین، روی هایپرلیپیدمی می باشد. مواد و روش ها: در این مطالعه که از نوع کارآزمایی بالینی بود سه گروه 25 نفری شامل افراد 40 تا 65 سال با هایپرلیپیدمی که ویژگیهای فردی از جمله وزن، نوع رژیم غذایی غالب، سابقه پزشکی، سطح فعالیت فیزیکی و سطح سرمی چربیهای خون مشابه داشتند، به طور تصادفی در سه گروه مورد مطالعه قرار گرفتند. قبل از مداخله درمانی، سطح سرمی چربیهای خون پایه در بیماران سنجش گردید. برای گروه اول، سرکه سیب با دوز 10 سی سی دوبار در روز، نیم ساعت قبل از نهار و شام به مدت چهار هفته و برای گروه دوم آبغوره با دوز مشابه و به مدت چهار هفته، برای گروه سوم قرص لواستاتین با دوز20 میلی گرم روزانه به مدت چهار هفته تجویزگردید. در حین درمان، بیماران از هیچگونه دارو یا ترکیب دیگری که روی مقادیر چربیهای خون تأثیر گذار باشد، استفاده نمی کردند. سطح سرمی چربیهای خون در پایان هفته دوم و هفته چهارم در هر سه گروه اندازه گیری شد و درنهایت اطلاعات بدست آمده با استفاده از نرم افزار آماری SPSS و آزمونهای آماری ANOVA، Bonferroni، TUKEY و همبستگی مورد تجزیه و تحلیل قرار گرفت. نتیجه گیری: سرکه سیب به عنوان جزء ساده ای از رژیم غذایی، در کاهش سطح چربیهای نامطلوب خون و درمان هایپرلیپیدمی تقریبا به اندازه لواستاتین مؤثر است. آبغوره نیز تا حدودی سطح کلسترول را کاهش می دهد ولی تأثیر آن به مراتب کمتر از سرکه سیب می باشد. یافته ها: در گروه تحت درمان با سرکه سیب، بین سطح سرمی کلسترول، LDL-C و HDL-C قبل از درمان،با سطوح سرمی آنها دو هفته و چهارهفته بعد از درمان، اختلاف معنی داری وجود داشت. در رابطه با گروه تحت درمان با لواستاتین و گروه تحت درمان با آبغوره نیز نتایج مشابه بدست آمد (001/0p<). میانگین سطح سرمی کلسترول دو هفته پس از درمان با سرکه،آبغوره و لواستاتین به ترتیب 16%، 5% و 21% و چهار هفته پس از درمان به ترتیب 25%، 8% و 26% کاهش یافته و HDL-C دو هفته پس از درمان به ترتیب 20%، 7% و 28% و پس از چهار هفته درمان به ترتیب 7/35%، 4/8% و 5/36% افزایش یافته بود. در هیچ یک از گروهها،تری گلیسرید پس از درمان کاهش معنی داری نداشت.

Title: مطالعه استریولوژیک اثر درازمدت اکتروتید (آنالوگ سوماتواستاتین) بر مهار گسترش مزانژیال گلومرولی در رتهای دیابتی – نفرکتومی یکطرفه

http://yafte.lums.ac.ir/article-1-1008-fa.html

مقدمه: نفروپاتی دیابتی یکی از علل مرحله نهایی بیماری کلیوی است. در دیابت قندی افزایش IGF-1 (فاکتور رشد شبه انسولینی –1) موضعی و افزایش هورمون رشد موجب القاء ضایعات کلیوی بویژه افزایش مزانژیوم داخل گلومرولی ودرنهایت اسکلروز گلومرولی میگردد. در این تحقیق تولید IGF-1 و هورمون رشد توسط اکتروتید مهار و تأثیر آن بر مهار اسکلروز گلومرولی بطور کمی بررسی گردید. مواد و روش ها: در این بررسی تجربی21 راس رت نر دوماهه نفرکتومی چپ گردیده و بطور تصادفی به سه گروه 7 تایی تقسیم شدند. با تزریق آلوکسان(mg/Kg 120 زیرجلدی) در گروههای دوم و سوم دیابت القاء گردید.5 روز بعد از القاء دیابت به گروه سوم مدت 8 هفته اکتروتید(10 میکروگرم در روز)بصورت زیر پوستی تزریق گردید، سپس تمام حیوانات نفرکتومی شده و کلیه برداشته شده و فیکس گردید. هر کلیه را به اسلایسهایی با ضخامت یک میلی متر بریده وبعداز پردازش بافتی از هر اسلایس برشی با ضخامت 5 میکرون تهیه و با روش PAS رنگ آمیزی شدند. با استفاده از روش کاوالیری و شمارش نقطه، حجم کورتکس کلیه، حجم گلومرولهاو حجم مزانژیوم برآورد گردید. تفاوت میانگین متغیرها با نرم افزار SPSS 12 و تست آماری Mann Withney در P<0.05 ارزیابی گردید. نتیجه گیری: مهار افزایش حجم کورتکس و مهار افزایش مزانژیوم درمان با اکتروتید بطور معنی داری نسبت به دیابتی بدون درمان مؤثرمی باشد.ولی این درمان نتوانسته است در مهار هیپرتروفی گلومرولی در رتهای دیابتی اثر معنی داری نشان دهد. یافته ها: مصرف اکتروتید با مقدار پایین و دراز مدت در رتهای دیابتی توانسته است نسبت به دیابتی های بدون درمان در جلوگیری از افزایش حجم کورتکس کلیه حدود 67% و در جلوگیری از افزایش حجم مزانژیوم حدود 53% بطور معنی دار مؤثر باشد. این درمان افزایش حجم گلومرولی را 26% مهار می کند.

Title: بررسی میزان شیوع رفتار های مخاطره آمیز بهداشتی در دانشجویان مراکز آموزش عالی شهر خرم آباد 84-1383

http://yafte.lums.ac.ir/article-1-1009-fa.html

مقدمه: رفتار های مخاطره آمیز بهداشتی عامل بروز بسیاری از این بیماریهای مزمن و حوادث است. شیوع اینگونه رفتارها بطور قابل ملاحظه ای به ویژه درمیان نوجوانان وجوانان درحال افزایش است. ازاین رومطالعه حاضرباهدف تعیین میزان شیوع رفتارهای مخاطره آمیزبهداشتی دردانشجویان مراکزآموزش عالی شهرخرم آباددرسال84-1383انجام شد. مواد و روش ها: در این مطالعه توصیفی ـ مقطعی تعداد 700 دانشجوی دانشگاههای لرستان، علوم پزشکی، جامع علمی کاربردی و دانشگاه آزاد شرکت نمودند. ابزار گرد آوری اطلاعات پرسشنامه ای مشتمل بر دو بخش خصوصیات دموگرافیک و 66 سئوال در مورد رفتار های بهداشتی بود. روایی و پایایی پرسشنامه به روش محتوایی و آزمون مجدد تأمین گردید. در هر دانشگاه پس از انتخاب کلاسها پرسشنامه بین کلیه دانشجویان کلاس توزیع و به روش خود ایفایی تکمیل گردید. نتیجه گیری: شیوع رفتار های مخاطره آمیز بهداشتی در دانشجویان ضرورت توجه به این قشر از جامعه و ارائه آموزشهای لازم در این زمینه را نشان می دهد. یافته ها: 1/67% دانشجویان شرکت کننده در مطالعه زن و 6/87% مجرد، با میانگین سنی 26/21 سال بودند. در خصوص رفتار های مربوط به ایمنی فردی و خشونت مشخص شد که 4/84% کسانی که سابقه موتور سواری داشتند هرگز از کلاه ایمنی استفاده نکرده و 1/44% در اتومبیل از کمربند استفاده نکرده اند. 9/13% سابقه حمل سلاح و 3/26% سابقه زد و خورد در یکسال گذشته را بیان نمودند. مصرف روزانه میوه، سبزی و لبنیات در رژیم بسیاری از دانشجویان وجود نداشت در حالیکه شیوع مصرف نوشابه و تنقلات کم ارزش بیش از 50 درصد بود. شیوع رفتار های غلط تغذیه ای در پسرها و دانشجویان خوابگاهی بیشتر بود (005/0p<). از نظر تحرک فیزیکی اکثریت دانشجویان زندگی کم تحرک داشتند که البته فعالیت فیزیکی پسر ها بهتر بود. 1/25% دانشجویان سابقه مصرف سیگار، 7/29% قلیان، 9% مشروبات الکلی، 3/8% مواد مخدر و 3/4% دارو های غیر مجاز را ذکر کرده بودند. شیوع مصرف این موارد در پسرها بیش از دختر ها بود (0001/0p=). در خصوص رفتار های پر خطرجنسی مشخص شد که 32% کسانی که سابقه مقاربت جنسی داشته اند دارای بیش از دو شریک جنسی بوده و 8/39% از هیچگونه وسیله محافظتی در هنگام مقاربت استفاده نکرده اند.

Title: بررسی رابطه بین اختلالهای روانی با سندرم روده تحریک پذیر (IBS)

http://yafte.lums.ac.ir/article-1-1010-fa.html

مقدمه: سندرم روده تحریک پذیر (IBS) اختلال شایعی است که باعث درد شکم، نفخ و اختلال در دفع مدفوع شده و می تواند همراه با ناتوانی های مهم و صرف هزینه های بالا باشد. مسائل روانی این سندرم بالا بوده و چیزی حدود 20 تا60% موارد با اختلالات روانی همراه، مرتبط است. اختلالات اضطرابی، اختلالات افسردگی و اختلالات شبه جسمی با فراوانی بیشتری توام با این سندرم گزارش شده اند. مواد و روش ها: روش مطالعه بصورت مقطعی ـ مقایسه ای و تعداد نمونه ها 76 نفر از بیماران با ناراحتی و مشکلات گوارش بود که طی مدت 6 ماه توسط متخصصان داخلی و گوارش تشخیص IBS برای آنها مطرح شده و با گروه کنترل مورد مقایسه قرار گرفتند. ابزار پژوهش شامل یک آزمون روانشناختی سلامت عمومی(GHQ-28)، یک چک لیست ملاکهای تشخیصی سندرم روده تحریک پذیر(Rome-II) و یک چک لیست ویژگیهای دموگرافیک بهمراه مصاحبه بالینی بود. نتیجه گیری: سندرم روده تحریک پذیر که یکی از شایعترین اختلالات عملکردی دستگاه گوارشی است، ارتباط زیادی با عوامل روانشناختی دارد. این پژوهش نشان میدهد که افسردگی، اضطراب و علائم جسمانی در این بیماران بیشتر است و توجه به مسائل روانشناختی و درمانهای غیر دارویی این بیماران با توجه به سبب شناسی روانی این سندرم توأم با درمانهای دارویی می تواند هم در کاهش علائم و هم در پائین آوردن هزینه های درمان این بیماری بسیار موثر واقع شود. یافته ها: نتایج پژوهش نشان داد میزان شیوع IBS در زنان، افراد متاهل، افراد جوانتر و افرادی که محل سکونت آنها در شهر بود، بیشتر است که این اختلاف از نظر آماری معنی دار است (05/0>p). اما این اختلاف در مورد میزان تحصیلات معنی دار نبود. همچنین اختلاف معنی داری بین گروه کنترل و IBS در میزان بروز افسردگی، اضطراب و علائم جسمانی بدست آمد، اما در مورد مقیاس کارکرد اجتماعی اختلاف معنی داری بدست نیامد،

Title: بررسی اثرات تعدیل‌کنندگی ایمنی غضروف کوسه ماهی در موش و انسان

http://yafte.lums.ac.ir/article-1-1011-fa.html

مقدمه: کوسه‌ها از موجوداتی هستند که به ندرت سرطان می‌گیرند. تفاوت اصلی بین این حیوانات و سایر حیوانات، وجود بافت غضروفی زیاد در بدن کوسه است. از آن‌جایی که سیستم ایمنی نقش مهمی در دفاع از بدن علیه برخی از انواع سرطان دارد، اثرات تعدیل کنندگی ایمنی غضروف کوسه ماهی بر سیستم ایمنی موش و انسان مطالعه گردید. مواد و روش ها: طی یک مطالعه تجربی، تأثیر دوزهای مختلف غضروف خالص کوسه بر تیتر آنتی‌بادی تولید شده علیه گلبول قرمز گوسفند (SRBC) در موش Balb/c بررسی شد. تأثیر غضروف بر فعالیت کشندگی سلول‌های کشنده طبیعی (NK) ، با تزریق این ترکیب به صفاق موش و سپس جداسازی سلول‌های صفاقی حیوان و میزان فعالیت کشندگی آنها علیه رده سلولی K562 بررسی شد. از تست ترانسفورماسیون لنفوسیتی (LTT) برای ارزیابی اثر غضروف کوسه بر فعالیت تکثیری سلول‌های تک هسته‌ای خون محیطی انسان بهره گرفته شد. بحث و نتیجه گیری: از آن جایی که غضروف کوسه می‌تواند سیستم ایمنی اختصاصی (سلولی و هومورال) را تقویت کند، از این ترکیب می‌توان برای تقویت و به طور کلی تعدیل پاسخ‌های ایمنی استفاده کرد و احتمالاً تعدیل پاسخ‌های ایمنی در برابر تومور فراهم کننده نوعی مکانیسم است که عامل ابتلای کمتر کوسه‌ماهی به سرطان می‌باشد. یافته ها: غضروف خالص کوسه، پاسخ ایمنی هومورال علیه آنتی‌ژن‌های موجود در گلبول قرمز گوسفند را در موش Balb/c تقویت کرد، به طوری که در دوزmg/kg 100، تیتر هماگلوتینین را به (در مقایسه با تیتر در حیوانات گروه شاهد که غضروف مرغ دریافت کرده بودند) افزایش داد. غضروف کوسه و مرغ هر دو دارای تأثیر میتوژنیک بر لنفوسیت‌های خون محیطی انسان هستند. غضروف خالص کوسه فعالیت NK را کاملاً سرکوب می‌کند.

Title: ارزیابی میزان بروز عوارض ناشی از واکسن در طرح ملی واکسیناسیون بر علیه سرخک و سرخجه در استان همدان

http://yafte.lums.ac.ir/article-1-1012-fa.html

مقدمه: در طی واکسیناسیون همگانی عوارض ناشی از تزریق اهمیت خاصی می یابد زیرا در عملیات تزریقی در سطح وسیع، عوارض افزایش یافته و به علت وسعت محدوده سنی احتمال بروزشان بالاتر است. این پژوهش جهت ارزیابی میزان بروز عوارض ناشی از واکسن در طرح ملی واکسیناسیون بر علیه سرخک و سرخجه در استان همدان انجام پذیرفت. مواد و روش ها: این مطالعه توصیفی مقطعی از 15 آذر تا 15 دی ماه 1382 و دو ماه پس از اتمام طرح، از طریق بررسی فرم های عوارض واکسن که در اختیار کلیه تیم های عملیاتی و مراکز بهداشتی- درمانی دولتی و خصوصی بود، انجام پذیرفت. اطلاعات پس از استخراج در برنامه Spss مورد تجزیه و تحلیل قرار گرفت. نتیجه گیری: این مطالعه نشان داد بدنبال واکسیناسیون کشوری MR در استان همدان میزان بروز عوارض خفیف بیش از عوارض شدید بوده و بیشترین میزان بروز عوارض در ارتباط با واکنش به واکسن گزارش شد و موردی از خطای برنامه دیده نشد، لذا برقراری سیستم مراقبت های سریع و انعطاف پذیری جهت عوارض جانبی پیشنهاد می گردد. یافته ها: در این مطالعه مشخص گردید 827468 نفر در استان همدان واکسینه و از این تعداد 260 نفر دچار عوارض واکسن شده اند، تعداد کل عوارض 605 مورد بود. 4/61% عوارض در 24 ساعت اول پس از واکسیناسیون و 91% عوارض سرپائی بودند. 84% عوارض توسط تیم عملیاتی و بیشترین میزان بروز عوارض در گروه سنی 14-10 سال گزارش شد. 86% بروز عوارض مربوط به واکنش به واکسن بود. عوارضی که میزان بروزشان بیشتر از یک مورد درصد هزار بود به ترتیب شامل کهیر، تب، سردرد، استفراغ، لنفاد نوپاتی، سرفه و راش، میالژی، فارنژیت، آرتریت، رینیت، بی قراری، عوارض چشمی و عوارضی که میزان بروزشان کمتر از یک مورد درصد هزار بود به ترتیب شامل تنگی نفس شدید، تشنج، اسهال، پارستزی، آنافیلاکسی، آنسفالیت، ترومبوسیتوپنی، آنسفالوپاتی و گیلن باره بودند.

Title: بررسی ذخایرآهن دربیماران کرونری بدون ریسک فاکتورشناخته شده

http://yafte.lums.ac.ir/article-1-1013-fa.html

مقدمه: بیماری عروق کرونریک بیماری شایع میانسالی به بعد است. مطالعات اپیدمیولوژیک قوی گواه اینست که آهن می تواند یک فاکتور مهم درایجاد بیماری عروق کرونر باشد. بنابراین مطالعه حاضر باهدف بررسی ارتباط بین وضعیت آهن وبیماری عروق کرونرانجام گردید. مواد و روش ها: شرکت کنندگان دراین مطالعه 208 نفردارای بیماری قلبی بودند. ابتدا مشخصات فردی آنان توسط پرسشنامه جمع آوری شد. سپس نمونه خون آنان جهت اندازه گیری آهن-فریتین و Hb-HCT-TIBC گرفته شد. بحث و نتیجه گیری: یافته ها نشان می دهد که با افزایش سن میزان ذخیره آهن بدن افزایش می یابد و این امر میتواند در بیماریزایی برخی بیماریهای شایع مثل بیماریهای قلبی-عروقی مؤثر باشد، بنابراین نتایج ما از نقش ذخایرآهن بعنوان یک عامل خطر در ایجاد بیماری عروق کرونر حمایت میکند. یافته ها: 110 نفر (9/52%) از شرکت کنندگان زن و 98 نفر (1/47%)آنان مرد بودند. میانگین سنی آنان 5/63 سال بود. میانگین سطح فریتین آقایان 041/127 و 214/108 میکروگرم درمیلی لیتر در خانم ها بود. میانگین سطح آهن بدن مردان 70/93 و در خانمها 71/84 میکروگرم در میلی لیتر بود. نتایج نشان داد که بین سن و سطح آهن و فریتین ارتباط وجوددارد

Title: بررسی پلی‌مورفیسم آنتی‌ژن پلاکت انسانی-1 (Human Platelet Antigene-1) در مبتلایان به آسم برونشیال

http://yafte.lums.ac.ir/article-1-1014-fa.html

مقدمه: آسم، نوعی بیماری التهابی و مزمن مجاری هوایی است که با افزایش پاسخدهی مجاری تراکئوبرونکیال، مشخص می‌شود. التهاب مزمن مجاری تنفسی در این بیماری ممکن است منجر به بروز تغییراتی در ساختار راههای هوایی گردد که به این تغییرات Airway Remodeling اطلاق می شود. فعال شدن پلاکتها در Remodeling راههای هوایی بیماران مبتلا به آسم نقش دارد. نظر به نقش پلاکت‌ها در Remodeling دیواره راههای هوایی و پیامدهای مشخص بروز Remodeling راههای هوایی در آسم، تعیین فراوانی آللهای مربوطه اهمیت ویژه ای دارد. بنابراین مطالعه حاضر با هدف بررسی پلی‌مورفیسم آنتی‌ژن پلاکتی انسانی-1 (HPA-1) در بیماران مبتلا به آسم، طراحی و انجام شد. مواد و روش ها: این مطالعه از نوع تحلیلی مورد- شاهدی بر روی 110 بیمار مبتلا به آسم انجام شد.گروه شاهد نیز 129 نفر بودند که از افراد با شکایات غیر تنفسی مراجعه کننده به درمانگاه داخلی بیمارستان اکباتان انتخاب شدند.سپس 10 میلی لیتر خون حاوی ماده ضد انعقاد از افراد بیمار و افراد گروه شاهد جهت تعیین فراوانی آلل‌های آنتی‌ژن پلاکت انسانی-1 (Ia, Ib) به آزمایشگاه ارسال شد. پلی‌مورفیسم HPAIa,b با روش pcr-ssp مورد بررسی قرار گرفت. اطلاعات جمع‌آوری شده توسط ویرایش دهم نرم‌افزار آماری SPSS، مورد تجزیه و تحلیل قرار گرفتند. نتیجه گیری: بررسی پلی‌مورفیسم ژن آنتی‌ژن پلاکتی انسان-1 (HPA-I) نشان داد که این ژن، نقشی در ایجاد آسم ندارد، ولی وجود آلل Ia می‌تواند با درجات شدیدتر آسم همراه باشد. یافته ها: بیشترین شکایات بیماران بعد از تنگی نفس، به ترتیب حملات تکرار شونده خس‌خس سینه (6/93%)، سرفه‌های مکرر(90%) و تشدید سرفه یا تنگی نفس در شب (1/89 درصد) بود.توزیع فراوانی آلل‌های Ia و Ib و ژنوتیپ‌های Ia+Ia، Ia+Ib و Ib+Ib آنتی‌ژن پلاکت انسانی-1، در دو گروه مورد و شاهد بررسی شد. هیچ موردی از ژنوتیپ Ib+Ib در دو گروه مورد وشاهد مشاهده نشد. نتایج این مطالعه نشان داد که توزیع فراوانی آلل Ia و Ib و نیز توزیع فراوانی ژنوتیپ‌های موجود Ia+Ia و Ia+Ib در دو گروه مورد و شاهد یکسان بود. میزان فراوانی آلل Ia و ژنوتیپ Ia+Ia با شدت آسم نسبت مستقیم داشت و هرچه آسم شدیدتر می شد، فراوانی آلل Ia و ژنوتیپ Ia+Ia بیشتر بود.

Title: بررسی تأثیر رژیم درمانی استروژن در درمان بی اختیاری ادرار استرسی در مقایسه با ایمی پرامین در بانوان یائسه مراجعه کننده به مطب های شهر خرم آباد

http://yafte.lums.ac.ir/article-1-1015-fa.html

مقدمه: خانمها بیش از یک سوم عمر خود را در دوران یائسگی سپری می کند و بیش از 50% آنها با مشکلات ادراری تناسلی روبرو هستند، شایع ترین نوع بی اختیاری ادرار در خانمها بی اختیاری ادرار استرسی می باشد و خانمهای میانسال پراهمیت ترین گروهی می باشند که به این مشکل مبتلا هستند، این مشکل می تواند اثرات منفی بر کیفیت زندگی زنان یائسه چه از نظر روانی و چه از نظر جسمی داشته باشد. بحث و نتیجه گیری: نتایج بدست آمده نشان می دهد که ایمی پرامین در درمان بی اختیاری ادرار استرسی مؤثرتر از استروژن می باشد و درمان ترکیبی با ایمی پرامین و استروژن مؤثرتر از مصرف ایمی پرامین به تنهایی نیست. یافته ها: درصد بهبودی در گروه اول که استروژن دریافت کرده بودند، 5/22% معادل 7 نفر بود. درصد بهبودی در گروه دوم که ایمی پرامین مصرف کرده بودند، 8/54% معادل 17 نفر بود. درصد بهبودی در گروه سوم که هر دو دارو را با هم مصرف نمودند 9/70% معادل 22 نفر بود. مواد و روش ها: ما در این مطالعه 93 خانم یائسه مبتلا به SUI را به مدت سه ماه تحت درمان قرار دادیم. سه گروه 31 نفره وجود داشت. به گروه اول استروژن، به گروه دوم ایمی پرامین و به گروه سوم استروژن و ایمی پرامین به صورت ترکیبی دادیم. مطالعه انجام شده، یک مطالعه کارآزمایی بالینی است و هدف اصلی آن بررسی تأثیر استروژن در درمان بی اختیاری ادرار استرسی در مقایسه با ایمی پرامین در بانوان یائسه می باشد. روش جمع آوری اطلاعات در این مطالعه پرسشنامه می باشد.

Title: تأثیر احتمالی سمیت سلولی عنصرروی بر سلولهای رده لنفوئیدی راجی (Raji) باتکنیک فلورسانس

http://yafte.lums.ac.ir/article-1-1016-fa.html

مقدمه: عنصر روی (Zn) درحفظ سلامتی انسان ودر ساختار و عملکرد سیستم ایمنی نقش بارزی دارد. این مطالعه به منظور بررسی تاثیر احتمالی سمیت سلولی عنصر روی بر سلولهای- B (راجی) در شرایط آزمایشگاهی انجام شد. نتیجه گیری: ترکیبات روی بررده سلولی راجی دارای اثر سمیت سلولی وابسته به دوز بوده واحتمالاً بتوان از آن در آینده در تنظیم سیستم ایمنی استفاده نمود. یافته ها: آنالیز داده ها نشان داد که بین میزان زنده ماندن و رشد سلول ها در گروههای آزمون و شاهد تا غلظت‌ 100میکرومولار در ساعات 12 تا 72 اختلاف معنی داری وجود ندارد؛ اما در 200میکرومولار تا 500 میکرومولار بعد از 12، 24 و .... ساعت انکوباسیون ,این پارامترها درگروه آزمون نسبت به گروه شاهدکاهش معنی دار یافت (05/0p<). مواد و روش ها: این مطالعه تحلیلی-توصیفی وبنیادی با استفاده از تکنیک کشت سلولی رده لنفوئیدی راجی در شرایط آزمایشگاهی در مجاورت غلظت های مختلف روی در زمانهای متفاوت (12تا72 ساعت) در دمای 37درجه سانتیگراد و5 درصد CO2 انکوبه گردید. آنگاه میزان زنده ماندن ورشد سلول ها با رنگ آمیزی فلورسانس ) 10 میکرو لیتر از مخلوط اکریدین اورنج و اتید یوم برو ماید) بررسی شد.

Title: بررسی حساسیت و ویژگی دیلاتاسیون و کورتاژ در تشخیص خونریزی غیر طبیعی رحمی

http://yafte.lums.ac.ir/article-1-1017-fa.html

مقدمه: خونریزی رحمی غیرطبیعی (AUB) یکی از شایعترین مشکلات پزشکی در قلمرو بیماریهای زنان محسوب می‌شود. یکی از روشهای تشخیص انجام دیلاتاسیون و کورتاژ (D&C) می‌باشد که براساس نتایج حاصل از آن ممکن است اقدامات درمانی قطعی‌تر همچون هیسترکتومی صورت گیرد. هدف از این مطالعه، تعیین حساسیت و ویژگی D&C در AUB بود. مواد و روش ها: در این مطالعه توصیفی نتایج هیستوپاتولوژیک حاصل از D&C و هیسترکتومی با هم مقایسه شدند. تعداد 85 بیمار مبتلا به AUB که تحت D&C و سپس هیسترکتومی (بدلیل عدم پاسخ به درمان طبی یا گزارش پاتولوژیست دال بر پاتولوژی آندومتر غیرقابل درمان طبی) قرار گرفته بودند، وارد مطالعه شدند. مطالعه در بیماران مبتلا به AUB مراجعه کننده به بیمارستان بعثت در طـی سالهای 1381 لغایت 1383 (سه سال) صـورت گرفت. داده های لازم با استفاده از مشاهدات پاتولوژیست، نتایج مربوط به D&C و هیسترکتومی به دست آمده و در چک‌لیست ثبت می شد. این داده ها با استفاده از آمار توصیفی، تست x2 و نرم افزار SPSS تجزیه و تحلیل شد. نتیجه گیری: براساس نتایج این بررسی D&C روشی نسبتاً بی‌خطر و ارزان و در دسترس برای بررسی اغلب موارد AUB می‌باشد. توصیه می‌شود در مواردی که علیرغم وجود تخمک‌گذاری، خونریزی غیرطبیعی ادامه دارد و یا در موارد شک بالینی به ضایعات آندومتریال، هیستروسکپی مدنظر باشد. یافته ها: نتایج مطالعه نشان داد 3/75% بیماران ساکن روستا و 6/70% آنها بی‌سواد بودند. اغلب بیماران، سه حاملگی یا بیشتر را تجربه کرده بودند و فقط 4/2% در گروهی قرار داشتند که کمتر از 3 حاملگی داشتند. میانگین سنی بیماران 25/47 سال بود، حداقل سن 26 و حداکثر 78 سال بود. روش پیشگیری از حاملگی در اکثریت بیماران مورد مطالعه در زمان هیسترکتومی بستن لوله‌های رحمی بود (2/41%). علت هیسترکتومی در حدود 1/94% بیماران ادامه خونریزی غیرطبیعی علیرغم درمان طبیعی بود. حساسیت روش D&C در تشخیص خونریزی غیر طبیعی رحمی 26/78 درصد و ویژگی آن 16/79 درصد بود.

Title: تعیین وفور نسبی جهش های ژن GJB2 در جمعیت ناشنوایان غیرسندرومی جسمی مغلوب استان لرستان

http://yafte.lums.ac.ir/article-1-1018-fa.html

مقدمه: ناشنوایی مادرزادی که علل ژنتیکی و محیطی بسیاری دارد، یک از هر هزار نوزاد تازه متولد شده را تحت تأثیر قرار می‌دهد. جهش های ژن GJB2 که رمز کننده کانکسین 26 پروتئین تشکیل دهنده اتصال باز است به عنوان اساس ناشنوایی غیرسندرومی جسمی مغلوب شناخته شده است. مواد و روش ها: هدف ما در این مطالعه تعیین وفور نسبی جهش های ژن GJB2 در جمعیت ناشنوای غیرسندرومی جسمی مغلوب استان لرستان با استفاده از تکنیک DHPLC، ARMS، PCR و تعیین توالی مستقیم بود. یافته ها: در این مطالعه 106 کروموزوم (از 53 فرد بیمار) مورد بررسی قرار گرفت، 18 کروموزوم (17%) در ژن GJB2 جهش داشتند. جهش 35delG در ده کروموزوم (4/9%) (چهار بیمار هموزیگوت و دو بیمار هتروزیگوت بود) تشخیص داده شد. سایر جهش هایی که در این منطقه دیده شد، شامل: V95M، W24X، 3170G>A، 35delG 512insAACG، 314del14 و 510insCGAA بودند. همچنین پلی مورفیسم V153I در سه فرد بصورت هتروزیگوت دیده شد و آخرین جهش(510insCGAA) یک جهش جدید می باشد که وجود آن تاکنون در جمعیتهای دیگر جهان گزارش نشده است، نتیجه گیری: نتایج این مطالعه در مقایسه بافراوانی جهش ژن کانکسین 26که عامل بیش از 50% ناشنوایی های غیر سندرمی در جمعیتهای مختلف جهان می باشد، بسیار اندک بوده و فقط 17% موارد را شامل می شود، ازطرفی دراین مطالعه جهش( 510insCGAA) یک جهش جدید می باشد که وجود آن تا کنون در جمعیتهای دیگر جهان گزارش نشده است. این نتایج نشان می دهد که در جمعیت ما احتمالا" ژن های دیگری عامل ایجاد ARNSHL می باشد.
